# Race, Age, and Kidney Transplant Waitlisting Among Patients Receiving Incident Dialysis in the United States

**DOI:** 10.1016/j.xkme.2023.100706

**Published:** 2023-08-05

**Authors:** Jade Buford, Samantha Retzloff, Adam S. Wilk, Laura McPherson, Jessica L. Harding, Stephen O. Pastan, Rachel E. Patzer

**Affiliations:** 1 Regenstrief Institute, Indianapolis, Indiana; 2 HIV Surveillance Branch (HSB), Division of HIV Prevention (DHP), National Center for HIV/AIDS, Viral Hepatitis, STD, and TB Prevention (NCHHSTP), Centers for Disease Control and Prevention, Atlanta, Georgia; 3 Department of Health Policy and Management, Rollins School of Public Health, Emory University, Emory University School of Medicine, Atlanta, Georgia; 4 Department of Epidemiology, Rollins School of Public Health, Emory University, Emory University, Emory University School of Medicine, Atlanta, Georgia; 5 Division of Transplantation, Department of Surgery, Emory University, Emory University School of Medicine, Atlanta, Georgia; 6 Health Services Research Center, Emory University School of Medicine, Emory University, Emory University School of Medicine, Atlanta, Georgia; 7 Department of Medicine, Renal Division, Emory University, Emory University School of Medicine, Atlanta, Georgia; 8 Department of Surgery, Indiana University School of Medicine, Indianapolis, Indiana

**Keywords:** Kidney failure, patients with kidney failure, transplant, waitlisting, access to care, disparities, race/ethnicity, age

## Abstract

**Rationale & Objective:**

Patients with kidney failure from racial and ethnic minority groups and older patients have reduced access to the transplant waitlist relative to White and younger patients. Although racial disparities in the waitlisting group have declined after the 2014 kidney allocation system change, whether there is intersectionality of race and age in waitlisting access is unknown.

**Study Design:**

Retrospective cohort study.

**Setting & Participants:**

439,455 non-Hispanic White and non-Hispanic Black US adults initiating dialysis between 2015 and 2019 were identified from the United States Renal Data System, and followed through 2020.

**Exposures:**

Patient race and ethnicity (non-Hispanic White and non-Hispanic Black) and age group (18-29, 30-49, 50-64, and 65-80 years).

**Outcomes:**

Placement on the United Network for Organ Sharing deceased donor waitlist.

**Analytical Approach:**

Age- and race-stratified waitlisting rates were compared. Multivariable Cox proportional hazards models, censored for death, examined the association between race and waitlisting, and included interaction term for race and age.

**Results:**

Over a median follow-up period of 1 year, the proportion of non-Hispanic White and non-Hispanic Black patients waitlisted was 20.7% and 20.5%, respectively. In multivariable models, non-Hispanic Black patients were 14% less likely to be waitlisted (aHR, 0.86, 95% CI, 0.77-0.95). Relative differences between non-Hispanic Black and non-Hispanic White patients were different by age group. Non-Hispanic Black patients were 27%, 12%, and 20% less likely to be waitlisted than non-Hispanic White patients for ages 18-29 years (aHR, 0.73; 95% CI, 0.61-0.86), 50-64 (aHR, 0.88; 95% CI, 0.80-0.98), and 65-80 years (aHR, 0.80; 95% CI, 0.71-0.90), respectively, but differences were attenuated among patients aged 30-49 years (aHR, 0.89; 95% CI, 0.77-1.02).

**Limitations:**

Race and ethnicity data is physician reported, residual confounding, and analysis is limited to non-Hispanic White and non-Hispanic Black patients.

**Conclusions:**

Racial disparities in waitlisting exist between non-Hispanic Black and non-Hispanic White individuals and are most pronounced among younger patients with kidney failure. Results suggest that interventions to address inequalities in waitlisting may need to be targeted to younger patients with kidney failure.

**Plain-Language Summary:**

Research has shown that patients from racial and ethnic minority groups and older patients have reduced access to transplant waitlisting relative to White and younger patients; nevertheless, how age impacts racial disparities in waitlisting is unknown. We compared waitlisting between non-Hispanic Black and non-Hispanic White patients with incident kidney failure, within age strata, using registry data for 439,455 US adults starting dialysis (18-80 years) during 2015-2019. Overall, non-Hispanic Black patients were less likely to be waitlisted and relative differences between the two racial groups differed by age. After adjusting for patient-level factors, the largest disparity in waitlisting was observed among adults aged 18-29 years. These results suggest that interventions should target younger adults to reduce disparities in access to kidney transplant waitlisting.

## Graphical abstract

**Figure:**
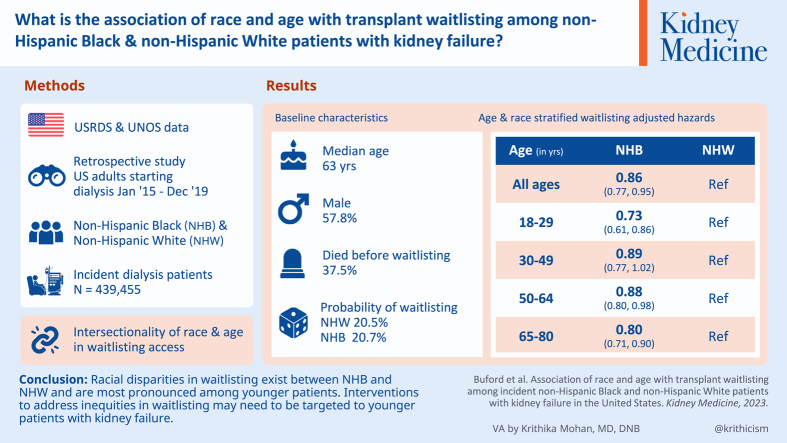

Racial discrimination and bias have been longstanding within the healthcare system, directly affecting patient access and satisfaction of care, including access to kidney transplantation among patients with kidney failure.^1^ In 2019, the number of patients with kidney failure in the United States exceeded 800,000, with the prevalence > 4 times greater among Black patients compared with White patients.^2^ Kidney transplantation results in significantly better survival, quality-of-life, and economic benefits compared to indefinite dialysis for people with kidney failure;^3^^,^^4^ however, access is not equitable.^2^^,^^4^ Racial disparities exist at all steps of the kidney transplant process, including referral, completion of evaluation, waitlisting, and receipt of a deceased or living donor kidney transplant.^5^, ^6^, ^7^, ^8^, ^9^, ^10^ Furthermore, some evidence suggests an intersection between race and age.^2^^,^^11^ For example, Black versus White patients with kidney failure receiving dialysis have higher survival among older individuals, but lower survival among younger individuals.^12^ Similarly, racial disparities in both transplantation and home dialysis 90 days after kidney failure treatment initiation are more prominent among younger than older adults.^13^

In December 2014, the 2014 Kidney Allocation System (KAS) was implemented. KAS was designed to reduce racial disparities in access to deceased donor kidney transplantation by redefining the start of waiting time from the time of waitlisting to the time of kidney failure treatment initiation. Early data suggests declines in racial disparities in waitlisting^14^ and transplant receipt^15^ after KAS, although longer-term data is needed. Post-KAS data has also shown decreases in waitlisting and transplantation among older, but not younger, patients.^16^ However, whether the impact of race on waitlisting varies with age in the post-KAS era remains unknown. Therefore, in this study, we examine whether age modifies racial disparities in kidney transplant waitlisting.

## Methods

### Data sources and study population

**Figure 1:**
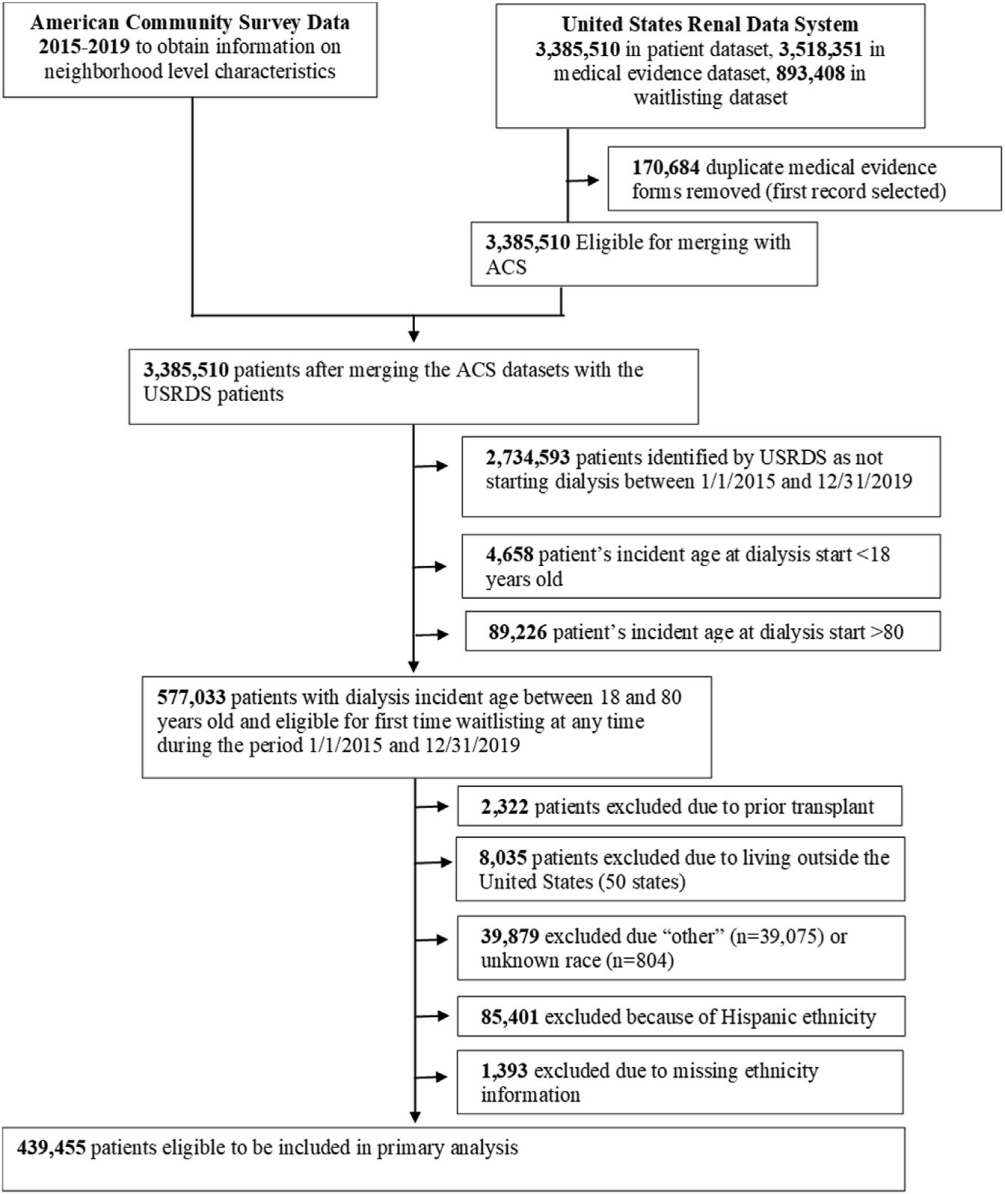
Data merge and cohort selection to examine the relationship between race and age on waitlisting on the national transplant waitlist.

We identified all adult (18-80 years) patients with kidney failure initiating dialysis between January 1, 2015, and December 31, 2019 (n=577,033), from the United States Renal Data System (USRDS)^2^ and included follow-up through December 31, 2020.^2^ Patients >80 years were excluded (n=89,226) given lower waitlisting among older patients due to higher comorbidity burden and increased risk of poor posttransplant outcomes.^17^^,^^18^ Patients identified as having a previous transplant (n=2,870), “other” (n=39,075) or unknown (n=804) race, Hispanic ethnicity (n=85,401), those missing ethnicity information (n=1,393), and those residing outside the 50 US states at time of start of dialysis (n=8,035) were excluded. The final analytic sample included 439,455 incident-US adult patients with kidney failure receiving kidney replacement therapy and identifying as non-Hispanic Black or non-Hispanic White (Figure 1).

Patient information was obtained from the USRDS patient data, Medical Evidence Form (CMS-2728) data, and the USRDS data on waitlisting events obtained from the United Network for Organ Sharing (UNOS) data on kidney transplantation waiting list and transplant events. Given that some patients had multiple CMS Medical Evidence Forms, the first record providing information on comorbidity status at kidney replacement therapy (KRT) initiation was used. Outcome data on waitlisting was obtained from the UNOS data contained within the USRDS waitlisting dataset. Neighborhood-level characteristics were determined by linkage to the 2015-2019 American Community Survey using the patients’ 5-digit ZIP code. Dialysis facility-level information was obtained from the USRDS facility dataset. Sources of data within this file are the CMS end-stage renal disease Annual Facility Survey and the Centers for Disease Control and Prevention Dialysis Surveillance Survey collected between January 1, 2018, and December 31, 2018. This data was linked to patient observations from the USRDS using the USRDS Assigned Facility ID. This study was exempted from review and a waiver of informed consent was granted for this retrospective, deidentified study by the Emory Institutional Review Board.

### Study Variables

The primary outcome was placement on the UNOS waiting list for a deceased donor kidney (ie, waitlisting). Study participants were identified at the initiation of dialysis and followed until waitlisting, death, or the end of the study (December 31, 2020), whichever occurred first. Race and age were the primary exposures in the analysis. Race was defined as non-Hispanic White or non-Hispanic Black. Here, we consider race a social construct, not a biologic categorization.^19^ Age groups were created *a priori* by mirroring the age stratification used in Kucirka et al^12^ and separating the group of patients aged 50-80 years to account for known low waitlisting rates, lack of access to other steps in the transplantation process, and low transplantation rates among patients >65 years.^12^^,^^20^ Age was stratified into 4 groups: 18-29, 30-49, 50-64, and 65-80 years. Patient-level characteristics included age, sex, race, body mass index, attributed cause of kidney failure (diabetes, hypertension, glomerulonephritis, polycystic kidney, urologic conditions, other causes, and unknown causes), year of start of KRT, and comorbidities at the time of start of kidney failure (atherosclerotic heart disease, cardiac failure, peripheral vascular disease, cerebrovascular disease, hypertension, diabetes, tobacco use, cancer, chronic obstructive pulmonary disease, drug abuse, alcohol abuse, and neighborhood poverty). Socioeconomic indicators included primary health insurance and access to pre-kidney failure nephrology care (yes/no). Neighborhood poverty was defined as ≥20% (or <20%) of households living below the poverty level.

### Statistical Analyses

Descriptive statistics were calculated for patient- and neighborhood-level factors for the study population and stratified by race and age. The cumulative incidence estimated probabilities of placement of patients with kidney failure on the waitlist during follow-up by race and age, treating death as a competing risk using the model of Fine and Gray.^21^ The 95% confidence intervals (95% CIs) were calculated with robust variance estimates.

We used crude and multivariable-adjusted Cox proportional hazards models, adjusted for patient demographic and clinical characteristics and censored for death,^12^ to assess the association between race and waitlisting. Demographic and clinical characteristics were considered for potential inclusion in the model *a priori* if they had previously been shown to be associated with race and/or age, or waitlisting.^5^, ^6^, ^7^, ^8^^,^^22^, ^23^, ^24^, ^25^, ^26^ We used robust sandwich covariance matrix estimates to account for clustering of patients within dialysis facilities,^26^^,^^27^ and included an interaction term for age (likelihood ratio test for interaction, *P* < 0.001). After a significant interaction, we stratified all analyses by age. Given our large sample size, the proportional hazards assumption was tested by examining log-log curves, with no evidence of non-proportionality. To avoid selection bias, we included patients who were preemptively waitlisted (waitlisted before the start of dialysis), and their time to waitlisting was coded as 1 day. A complete case analysis, omitting cases with missing data, was used to account for the presence of missing patient data for explanatory variables included in the model. Less than 10% of the data was missing for all variables, excluding dialysis facility patient-to-social worker ratio information (15.75% missing).

Given high race-based disparities in young adults, we conducted additional subgroup analyses to examine the association between race and waitlisting among adults aged 18-29 years stratified by sex, insurance type, body mass index, pre-kidney failure nephrology care, primary cause of kidney failure, and percentage of neighborhood poverty subgroups to determine whether racial disparities in young adults were specific to patients with certain demographic characteristics or comorbidities. Crude and multivariable-adjusted Cox proportional hazards models, censored for death, were repeated within each subgroup. Additionally, sensitivity analyses were conducted to evaluate racial differences in the placement on the waitlist, accounting for death as competing events using Fine and Grey models to calculate the adjusted subdistribution hazard ratios. In a sensitivity analysis, descriptive statistics were calculated for patient- and neighborhood-level factors for patients who were preemptively waitlisted in comparison to those who were not, and evaluated the impact of including these preemptively waitlisted patients on differences in the incidence of waitlisting by race and age during the study period by obtaining the cumulative incidence estimated probabilities, excluding patients who were preemptively waitlisted. SAS 9.4 (SAS Institute Inc, Cary, NC) was used for data management and analyses.

## Results

### Study Population Characteristics

Among 439,455 patients with incident kidney failure with a median age 63.0 years (interquartile range [IQR], 53.0-71.0, 57.8% men), there were approximately twice as many non-Hispanic White patients (n = 289,217; 65.8%) compared to non-Hispanic Black patients (n = 150,238; 34.2%). Non-Hispanic White (vs non-Hispanic Black) patients were more likely to have private insurance and diabetes as the underlying cause of kidney failure. In comparison, non-Hispanic Black (vs non-Hispanic White) patients were more likely to have hypertension as the most frequently attributed causes of kidney failure and a higher percentage of patients with ZIP codes containing ≥20% of residents below the poverty. Racial differences in population characteristics persist across all age groups. The percentage of patients receiving pre-kidney failure nephrology care increased with age, with care less common among patients aged 18-29 years. Other comorbidities were similar between non-Hispanic White and non-Hispanic Black patients with kidney failure being common for all ages (Table 1; Table S1).

**Table 1:** Baseline Characteristics of Incident US Adult Patients With Kidney Failure (2015-2019) by Age and Race (N=439,455).

	Overall	Non-Hispanic White (n=289,217)	Non-Hispanic Black (n=150,238)
**Patient-level characteristics**			
Sex, n (%)			
Male	254,191 (57.8)	172,854 (59.8)	81,337 (54.1)
Female	185,264 (42.2)	116,363 (40.2)	68,901 (45.9)
Age, n (%)			
18-29 y	10,548 (2.4)	5,689 (2.0)	4859 (3.2)
30-49 y	70,181 (16.0)	37,164 (12.9)	33017 (22.0)
50-64 y	155,385 (35.4)	97,190 (33.6)	58195 (38.7)
65-80 y	203,341 (46.3)	149,174 (51.6)	54167 (36.1)
Insurance type,^a^ n (%)			
Medicaid	106,149 (24.2)	56,387 (20.3)	49762 (34.2)
Medicare	176,367 (40.1)	130,094 (46.9)	46273 (31.8)
Private	86,341 (19.7)	58,320 (21.0)	28021 (19.2)
Other	35,675 (8.1)	23,427 (8.5)	12248 (8.4)
No coverage	18,293 (4.2)	8,979 (3.2)	9314 (6.4)
Body mass index,^b^ n (%)			
<18	11,942 (2.3)	7,358 (2.6)	4584 (3.1)
18-24.9	104,709 (23.8)	67,506 (24.0)	37203 (25.2)
25-29.9	115,720 (26.3)	76,605 (27.2)	39115 (26.5)
≥30	196,783 (44.8)	130,130 (46.2)	66653 (45.2)
Attributed cause of kidney failure,^c^ n (%)			
Diabetes	200,827 (45.7)	134,912 (47.7)	65915 (44.4)
Hypertension	127,736 (29.1)	69,679 (24.6)	58057(39.1)
Glomerulonephritis	32,455 (7.4)	23,114 (8.2)	9341 (6.3)
Polycystic kidney	13,031 (3.0)	11,060 (3.9)	1971 (1.3)
Urologic	6,292 (1.4)	5,440 (1.9)	852 (0.6)
Other	45,062 (10.3)	34,852 (12.3)	10210 (6.9)
Unknown	5,983 (1.4)	3,953 (1.4)	2030 (1.4)
Comorbidity, n (%)			
Hypertension	37,7857 (87.7)	242,497 (85.8)	135360 (91.3)
Diabetes	253,851 (58.9)	166,363 (58.9)	87488 (59.0)
Cardiac failure	122,460 (28.4)	80,925 (28.6)	41535 (28.0)
Tobacco use	35,245 (8.2)	23,301 (8.2)	11944 (8.1)
Drug abuse	7,241 (1.7)	3,581 (1.3)	3660 (2.5)
Atherosclerotic heart disease	54,455 (12.6)	41,608 (14.7)	12847 (8.7)
Peripheral vascular disease	40,699 (9.4)	30,039 (10.6)	10660 (7.2)
Cerebrovascular disease	38,589 (9.0)	23,871 (8.4)	14718 (9.9)
Other cardiac disease	87280 (20.25)	63,669 (22.5)	23611 (15.9)
Cancer	31,191 (7.2)	23,536 (8.3)	7655 (5.2)
Alcohol abuse	8,056 (1.9)	5,204 (1.8)	2852 (1.9)
Chronic obstructive pulmonary disease	44,635 (10.4)	33,839 (12.0)	10796 (7.3)
Pre-kidney failure nephrology care,^d^ n (%)			
No	80,793 (18.4)	48,582 (19.7)	32211 (26.2)
Yes	288,600 (65.7)	197,840 (80.3)	90760 (73.8)
Neighborhood poverty (ZIP code of residents below poverty),^e^^,^^f^ n (%)			
0%-19.9% below poverty	366,389 (83.4)	262,592 (91.8)	103797 (69.9)
≥20% below poverty	68,232 (15.5)	23,601 (8.3)	44631 (30.1)
**Dialysis facility-level characteristics**^g^			
Profit status,^h^ n (%)			
For profit	429,256 (97.8)	282,420 (97.8)	146836 (97.8)
Non-profit	5,124 (1.2)	3,381 (1.2)	1743 (1.2)
Patient-to-staff ratio, mean (SD)^i^	46.9 (7.5)	47.0 (6.9)	47.0 (8.5)

a Insurance information missing for 16630 patients (3.78%).

b Body-mass index calculated as weight in kilograms divided by height in meters squared, missing for 10301 patients (2.34%).

c Patient attributable cause missing for 8069 patients (1.84%).

d Nephrology care information missing for 70062 (15.94%).

e Obtained from American Community Survey Data, 2015-2019.

f Information on neighborhood poverty missing for 4834 (1.10%).

g Obtained from data from the CMS end-stage renal disease Annual Facility Survey and the Centers for Disease control and Prevention Dialysis Surveillance Survey within the United States Renal Data System facility dataset.

h Information on dialysis facility profit status missing for 495 patients (0.1%) and unknown for 4580 patients (1.0%).

i Number of patients for every 1 social worker. Information on dialysis facility patient-to-social worker ratio was missing for 69236 patients (15.75%). Calculated only among those facilities that have social workers and whose information on number of patients and social workers was not missing.

### Placement on the Deceased Donor Waitlist for Transplant by Race

**Figure 2:**
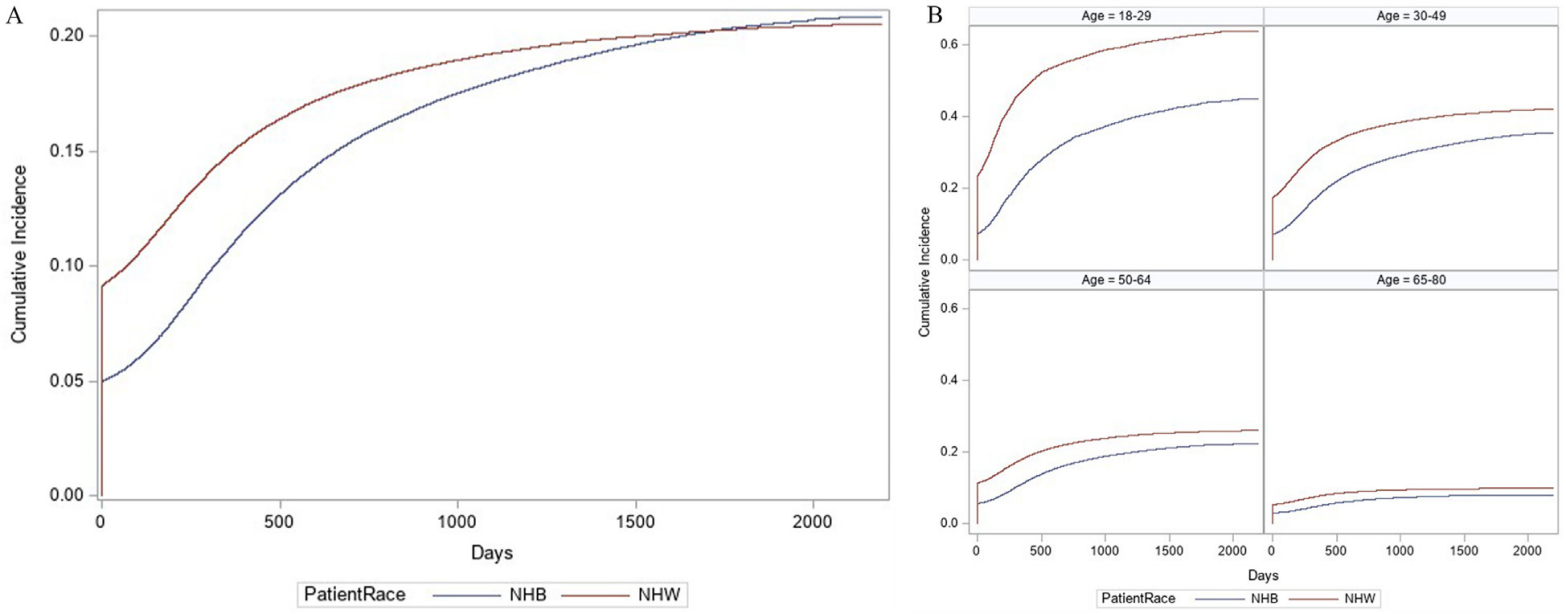
Cumulative incidence of waitlisting during the study period among (a) incident kidney failure patients by race and (b) incident kidney failure patients by race and age, censoring for death, 2015-2019, followed through 2020.^a,b^^a^Patients who were preemptively waitlisted (waitlisted before start of dialysis) were assigned a follow-up time of 1 day. ^b^Models were stratified because the effect of race (non-Hispanic Black and non-Hispanic White) on waitlisting differed by patient age (likelihood ratio test for interaction in adjusted analyses, p<0.001).

Overall, the probability of waitlisting for patients with kidney failure was 20.5% for non-Hispanic White and 20.7% for non-Hispanic Black patients, during a median follow-up of 588 (IQR, 201-1066; 1 year, IQR, 1-3) and 728 (IQR, 381-1265; 2 years, IQR, 1-3) days, respectively (Figure 2A, Table S2). About one-third (37.5%) of patients with kidney failure died before placement on the waitlist, including 30.4% non-Hispanic White and 41.2% non-Hispanic Black patients. In unadjusted analysis, non-Hispanic Black patients had lower rates of waitlisting than non-Hispanic White patients (HR, 0.86; 95% CI, 0.76-0.97) which remained similar in multivariable models (aHR, 0.86; 95% CI, 0.77-0.95; Table 2).

**Table 2:** Association Between Race and Waitlisting Among Incident Patients With Kidney Failure (2015-2019) by Age, Followed Through 2020.^a^

	Unadjusted HR (95% CI)	Adjusted HR (95% CI)^b^^,^^c^
All Ages	0.86 (0.76-0.97)	0.86 (0.77-0.95)
Age		
18-29 y	0.53 (0.43-0.66)	0.73 (0.61-0.86)
30-49 y	0.69 (0.59-0.81)	0.89 (0.77-1.02)
50-64 y	0.74 (0.65-0.83)	0.88 (0.80-0.98)
65-80 y	0.71 (0.62-0.81)	0.80 (0.71-0.90)

Abbreviations: HR, hazard ratio; CI, confidence interval.

a Non-Hispanic, White patients are reference group.

b Cox models were performed to obtain hazard ratios adjusting for age, sex, insurance type at kidney failure onset, body mass index, attributed cause of kidney failure (diabetes, hypertension, glomerulonephritis, polycystic kidney, urologic, unknown), atherosclerotic heart disease, cardiac failure, peripheral vascular disease, cerebrovascular disease, hypertension, diabetes, tobacco use, cancer, chronic obstructive pulmonary disease, drug abuse, alcohol abuse, neighborhood poverty.

c There was missing data for 82,239 patients (18.7%).

### Age-Stratified Analyses

By age, waitlisting was highest among patients aged 18-29 years (55.0% overall; 63.8% non-Hispanic White vs 44.7% non-Hispanic Black) and lowest in adults aged 65-80 years (9.3% overall; 9.8% non-Hispanic White vs 8.0% non-Hispanic Black) (Figure 2b and Table S3). Non-Hispanic Black patients aged 18-29 years had a higher risk of death compared with their non-Hispanic White counterparts. However, the risk of death was greater for non-Hispanic White vs non-Hispanic Black patients aged 30-49, 50-64, and 65-80 years (Table S2).

In multivariable models, race-based disparities in waitlisting differed by age group. Specifically, non-Hispanic Black patients were 27%, 12%, and 20% less likely to be waitlisted than non-Hispanic White patients for ages 18-29 (aHR, 0.73; 95% CI 0.61-0.86), 50-64 (aHR, 0.88; 95% CI 0.80-0.98), and 65-80 (aHR, 0.80; 95% CI, 0.71-0.90) years, respectively, but similarly likely to be waitlisted among patients 30-49 (aHR, 0.89; 95% CI 0.77-1.02) (Table 2).

### Subgroup Analyses Among Patients Aged 18–29-Years

Among patients with kidney failure aged 18-29-years, race disparities were consistent across sex, insurance status, body mass index, prekidney failure nephrology care (yes/no), primary cause of kidney failure ((hypertension, glomerulonephritis, polycystic kidney, other urologic conditions, and other causes), and neighborhood poverty (Item S1, Table S4).

### Sensitivity Analysis

In multivariable-adjusted subdistribution hazard models, adjusting for death as a competing risk, we found similar findings with the main analyses. Non-Hispanic Black patients had lower rates of waitlisting compared with non-Hispanic White patients in unadjusted (SHR, 0.92; 95% CI, 0.82-1.03) and multivariable models (aSHR, 0.88; 95% CI, 0.79-0.99, Table S5). In multivariable and age-stratified models, non-Hispanic Black patients were 26% and 15% less likely to be waitlisted than non-Hispanic White patients for ages 18-29 (aSHR, 0.74; 95% CI, 0.62-0.88) and 65-80 (aSHR, 0.85; 95% CI, 0.75-0.96) years, respectively, but the differences were attenuated among patients aged 30-49 (aSHR, 0.90; 95% CI, 0.78-1.04) and 50-64 (aSHR, 0.91; 95% CI, 0.83-1.01; Table S5) years. A total of 32,816 (7.5%) patients were preemptively waitlisted. Among these patients, 77.8% were non-Hispanic White and 22.2% were non-Hispanic Black. Among patients who were not preemptively waitlisting, there were 260,621 (64.1%) patients who received pre-kidney failure care. Non-Hispanic White patients who were not preemptively waitlisted had a larger percentage of patients aged 65-80 (53.6% vs 36.8%) years, a greater presence of atherosclerotic heart disease (15.2% vs 8.8%), and other cardiac diseases (23.2% vs. 16.1%) compared with non-Hispanic Black patients in this group, respectively. In comparison, among patients who were preemptively waitlisted, 26,979 (85.3%) received pre-kidney failure care. In addition, preemptively waitlisted patients were more likely to have private insurance (46.9%) and were aged 50-64 (42%) or 65-80 (28.5%) years (Table S6). After excluding preemptively waitlisting patients, incidence estimates showed, on average, consistent findings with main analyses where a lesser number of non-Hispanic White patients were waitlisted than non-Hispanic Black patients. Stratifying by age revealed racial disparities in waitlisting among patients aged 18-29 and 30-49 years, with non-Hispanic Black vs non-Hispanic White patients waitlisted less frequently. The incidence of waitlisting during the study period was higher for non-Hispanic Black patients aged 50-64 years (16.9% non-Hispanic White, 17.7% non-Hispanic Black) and similar between non-Hispanic White and non-Hispanic Black patients aged 65-80 years (4.8% non-Hispanic White, 5.2% non-Hispanic Black). (Item S2, Table S7, Figure S1).

## Discussion

In this study, we show that in the post-KAS era, racial disparities persist, and non-Hispanic Black patients with kidney failure are 14% less likely to be waitlisted compared with non-Hispanic White patients with kidney failure. Furthermore, we show this relative disparity is largest among younger adults, with non-Hispanic Black patients aged 18-29 years being 27% less likely to be waitlisted compared with their non-Hispanic White counterparts. Indeed, in this young age group, the race-based disparity is similar across sex, insurance type, body mass index, pre-kidney failure nephrology care, primary cause of kidney failure, and neighborhood poverty subgroups. These findings have important implications for the development of interventions to address racial disparities in access to kidney transplant waitlist. Specifically, we posit that interventions may have the most effect if they are targeted toward young Black patients with kidney failure.

Since implementation of KAS, there has been a 12%-19% decline in racial inequities in waitlisting in Black vs White patients.^14^ Despite this improvement, our research suggests that racial disparities persist. Causes of these inequities are multifactorial and multilevel and include poverty, insurance status, physician bias, medical mistrust, patient-perceived racism within the health care setting, structural racism,^28^ and discrimination.^6^^,^^7^^,^^24^^,^^25^^,^^29^ The lack of insurance, lower pre-kidney failure care, and higher poverty status that we observed among younger non-Hispanic Black (vs non-Hispanic White) patients with kidney failure may explain the reduced access to the waiting list.^6^^,^^7^^,^^13^^,^^24^^,^^25^^,^^30^ However, we found that these disparities persisted despite adjustment for these characteristics indicating that there are other factors causing this age difference we observed in racial disparities in waitlisting.

There are other unmeasured factors that may explain the observed racial disparities in waitlisting, especially among younger patients, including social networks, information about the transplant process, cultural/personal beliefs, structural racism, and patient preference.^24^^,^^31^, ^32^, ^33^, ^34^, ^35^, ^36^ Ayanian et al^31^ found that provider viewpoints regarding the survival advantage of transplantation by race, reasons for disparities, and patient preferences may influence communication of transplantation as a treatment offer.^31^ Recent research indicates that Black patients treated with hemodialysis are interested in receiving a transplant but are hindered by lack of communication from providers and limited knowledge of the transplantation process which influence perceptions of lack of interest.^32^, ^33^, ^34^, ^35^ Moreover, medical mistrust and attitudes of fear or suspicion toward medical institutions have been shown to be greater among younger (vs older) and non-Hispanic Black (vs non-Hispanic White) patients and may explain the greater disparity observed among younger patients.^1^^,^^37^ Furthermore, research has identified this medical mistrust, discrimination, and patient-perceived racism to be significantly associated with access to transplantation at the early steps in the transplant process, suggesting that these sociocultural factors should be considered as potential causes of exacerbated racial disparities in waitlisting, and robust solutions aimed at addressing racism at all levels of influence are needed.^25^^,^^29^

Prior research on disparities in the care of patients with kidney failure has suggested that older, but not younger, Black patients receiving dialysis have a survival advantage, similar to our findings. However, the mechanisms are unclear.^12^ Regardless, this survival advantage may contribute to our observed associations of waitlisting: older non-Hispanic Black patients may have a higher cumulative incidence of waitlisting than non-Hispanic White patients because they are less likely to die. The marginal increase in racial disparities in waitlisting among patients aged 65-80 years may be explained by studies showing the increased risk of complications after kidney transplant, which are higher among Black versus White patients. It may be reasonable to infer that these findings may have an impact on the selection of patients for placement on the waitlist and transplantation.^6^^,^^8^^,^^18^^,^^38^

Given the numerous upstream social determinants of health that influence racial disparities in kidney transplantation, it is important to focus interventions on these complex and multilevel factors which serve as the underlying fundamental causes of these inequities.^39^ Multicomponent interventions, including those at the health system–level, focused on improving education and quality of care within kidney transplant and dialysis centers nationally can improve equality in access,^36^ and prior successful interventions should be adapted to target this large racial disparity among young patients with kidney disease.^40^, ^41^, ^42^, ^43^, ^44^, ^45^, ^46^ Notably, interventions in HIV care focused on web-based delivery of information to reach young individuals have resulted in improved care engagement, including reducing barriers to care.^47^^,^^48^ Future research and interventions should consider the intersection between race and age to create lasting interventions to improve access to transplant among all patients with kidney failure.

This study has several limitations. First, there are potential factors that may be associated with placement on the waitlist, such as educational attainment, patient preferences, cultural/linguistic barriers, employment status, and other patient comorbidities, that were not measured in the current analysis.^6^, ^7^, ^8^^,^^24^^,^^25^ The magnitude of disparities between non-Hispanic Black and non-Hispanic White patients may be affected when adjusting for these factors; however, due to the large disparity we observed among young individuals with kidney failure, it is likely that this disparity would continue to exist, as other studies have found that racial disparities persist after accounting for many of these factors.^8^^,^^9^ Second, this analysis is limited to non-Hispanic Black and non-Hispanic White patients because of the larger population available for the subgroup analyses and continued evidence of inequities between Black and White patients with kidney failure. Future analysis should examine disparities in waitlisting among other subgroups. Third, this analysis does not consider disparities in living donor transplant. This should be explored in future analyses. Fourth, comorbidity data and information on patient race/ethnicity and other patient characteristics were captured in the CMS-2728 Medical Evidence Form at the time of start of kidney failure but may have been underreported or may have changed over time. Another notable limitation is the lack of information on physician-reported race/ethnicity by patients. The use of assigned race/ethnicity by clinicians or staff can be reflective of important exposures to discrimination that are not captured with self-reported race/ethnicity.^49^

In conclusion, this study provides evidence that in the post-KAS era, racial disparities in waitlisting persist between non-Hispanic Black and non-Hispanic White patients and these disparities are greatest in younger adults. Determining the reasons for greater disparities in waitlisting between non-Hispanic Black and non-Hispanic White patients, especially among the younger patients who experienced greater disparities, can help to inform future interventions and policies to improve equity in access to transplantation.

## Supplementary Material

Supplementary File (PDF) Figure S1. Item S1-S2. Table S1-S7.
