# Supplementary material for: Race, Age, and Kidney Transplant Waitlisting Among Patients Receiving Incident Dialysis in the United States
Source: Kidney Med. 2023 Aug 5;5(10):100706. doi: 10.1016/j.xkme.2023.100706 (PMC10518364; doi:10.1016/j.xkme.2023.100706)
Supplement: Supplementary File (PDF) — Figure S1. Item S1-S2. Table S1-S7. [file mmc1.pdf]

Figure S1. Incidence of Waitlisting During the Study Period Among A) Incident Kidney Failure Patients by Race and B) Incident Kidney Failure Patients by Race and Age, Excluding Preemptively Waitlisted Patients and Treating Death As a Competing Risk, 2015-2019, followed through 2020.

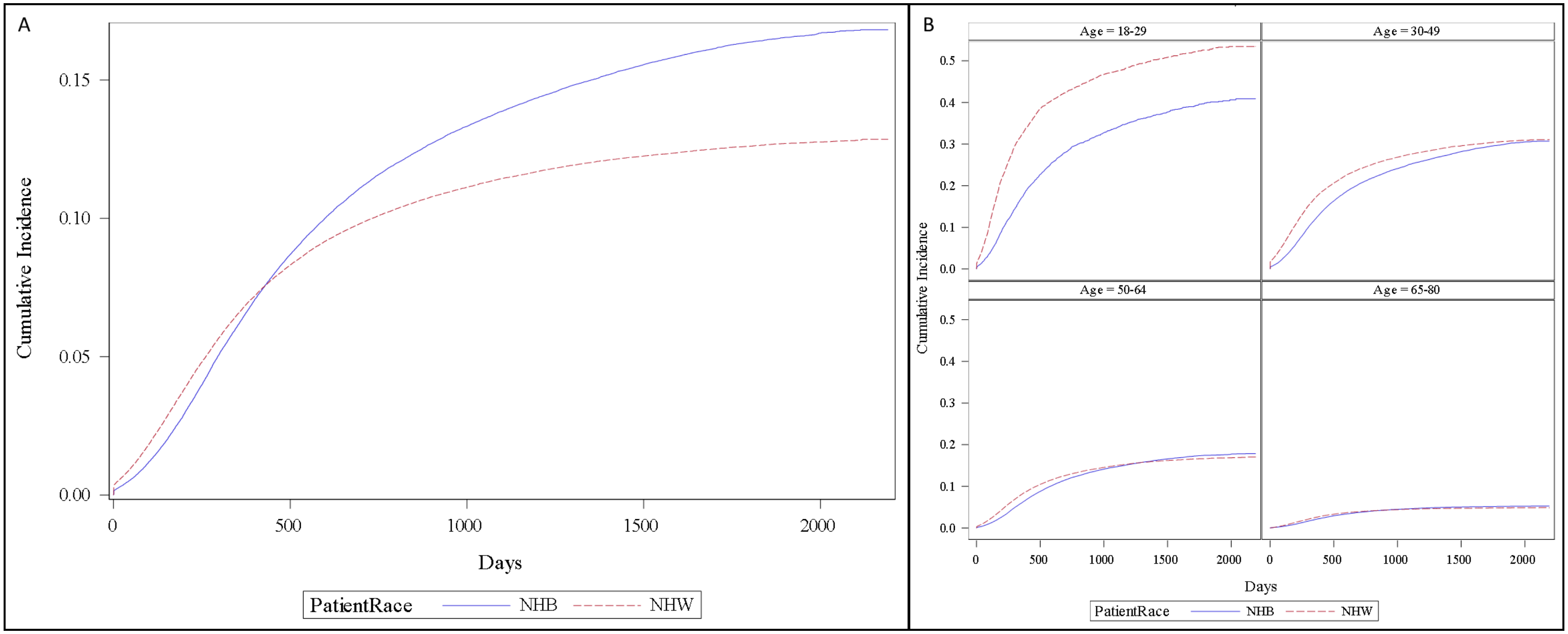

Item S1. Subgroup Analyses Among 18–29-Year-Olds

Multivariable-adjusted Cox proportional hazards models were repeated within different subgroups of 18–29-year-old patients based on demographic and clinical characteristics. Among 18–29-year-olds, non-Hispanic white patients were more likely to have private insurance, while a higher percentage of non-Hispanic Black patients had Medicaid (34.9% NHW vs 49.2% NHB), or no insurance coverage (9.2% NHW vs 15.9% NHB). In addition, hypertension was the attributed cause of kidney failure for a higher percentage of non-Hispanic Black patients (15.0% NHW vs 28.1% NHB). The percentage of patients with ZIP codes containing ≥20% of residents below the poverty line was higher among non-Hispanic Black patients (9.1% NHW vs 30.2% NHB). Other comorbidities were similar between non-Hispanic white and non-Hispanic Black kidney failure patients (**Table S1**).

The proportion of patients waitlisted within the various demographic and clinical groups demonstrates that these observed disparities in waitlisting between non-Hispanic Black and non-Hispanic white patients persist and are not specific to certain characteristics. Non-Hispanic Blacks had lower rates of waitlisting regardless of sex, body-mass index, pre- kidney failure care, percentage of neighborhood poverty, and attributed causes of kidney failure (hypertension, glomerulonephritis, polycystic kidney, other urologic conditions, and other causes **(Table S4)).**

Item S2. Sensitivity Analysis Excluding Preemptively Waitlisted Patients

The incidence estimated probabilities of kidney failure patients’ placement on the waitlist during follow-up by race and age, treating death as a competing risk was repeated excluding patients who were preemptively placed on the waitlist (waitlisted prior to dialysis start, n=32,816). Contrasting results found including patients who were preemptively waitlisted (time to waitlisting coded as one day), we found the incidence showed racial disparities in waitlisting between NHW (12.8%) and NHB patients (16.7%), with the incidence of waitlisting higher among NHB patients. Subgroup estimates of the proportion of patients waitlisted also indicate racial disparities in waitlisting, with NHB patients, being waitlisted less frequently compared to their NHW counterparts among the ages of 18-29 (53.4% NHW, 40.7% NHB,), 30-49 (31.0% NHW, 30.4% NHB). The incidence of waitlisting during the study period was higher for NHB patients aged 50-64 (16.9% NHW, 17.7% NHB) and similar between NHW and NHB patients aged 65-80 (4.8% NHW, 5.2% NHB). The median time from kidney failure start to waitlisting was approximately 654 days (IQR: 316-1123) for NHW patients and 771 days (IQR: 423-1297) for NHB patients **(Table S4, Figure S1a, and Figure S1b)**.

Table S1. Baseline Characteristics of Incident U.S. Adult Patients With Kidney Failure (2015-2019) by Age and Race[a] (N=439,455)

		Patient Age (Years)					
		18-29		30-49		50-64	
	Overall	Non-Hispanic White (n=5,689)	Non-Hispanic Black (n=4,859)	Non-Hispanic White (n=37,164)	Non-Hispanic Black (n=33,017)	Non-Hispanic White (n=97,190)	Non-Hispanic Black (n=58,195)
**Patient-Level Characteristics**							
Sex							
Male	254,191 (57.8)	3208 (56.4)	2363 (48.6)	22717 (61.1)	19495 (59.0)	59140 (60.8)	32845 (56.4)
Female	185,264 (42.2)	2481 (43.6)	2496 (51.4)	14447 (38.9)	13522 (41.0)	38050 (39.2)	25350 (43.6
Insurance type[b]							
Medicaid	106,149 (24.2)	1983 (34.9)	2392 (49.2)	12599 (33.9)	13395 (40.6)	25629 (26.4	21475 (36.9
Medicare	176,367 (40.1)	197 (3.5)	197 (4.1)	3828 (10.3)	3170 (9.6)	21423 (22.0)	11665 (20.0)
Private	86,341 (19.7)	1990 (35.0)	986 (20.3)	12686 (34.1)	8671 (26.3)	30144 (31.0)	13595 (23.4)
Other	35,675 (8.1)	750 (13.2)	431 (8.9)	3609 (9.7)	2682 (8.1)	11602 (11.9)	6194 (10.6)
No coverage	18,293 (4.2)	524 (9.2)	774 (15.9)	3107 (8.4)	4482 (13.6)	4752 (4.9)	3773 (6.5)
Body-mass index[c]							
<18	11,942 (2.3)	383 (6.7)	250 (5.1)	936 (2.5)	670 (2.0)	2183 (2.2)	1626 (2.8)
18-24.9	104,709 (23.8)	2327 (40.9)	1628 (33.5)	8727 (23.5)	6547 (19.8)	19962 (20.5)	13580 (23.3)
25-29.9	115,720 (26.3)	1231 (21.6)	1066 (21.9)	8806 (23.7)	7690 (23.3)	24039 (24.7)	14958 (25.7)
≥30	196,783 (44.8)	1489 (26.2)	1827 (37.6)	17330 (46.6	17477 (52.9	48188 (49.6	26958 (46.3)
Attributed cause of kidney failure[d]							
Diabetes	200,827 (45.7)	885 (15.6)	1169 (24.1)	15859 (42.7)	12591 (38.1	49081 (50.5)	26927 (46.3
Hypertension	127,736 (29.1)	856 (15.0)	1364 (28.1)	6984 (18.8)	13309 (40.3	19711 (20.3)	21850 (37.5
Glomerulonephritis	32,455 (7.4)	2030 (35.7)	1531 (31.5)	5248 (14.1)	3286 (10.0)	7351 (7.6)	2888 (5.0)
Polycystic kidney	13,031 (3.0)	544 (9.6)	103 (2.1)	3168 (8.5)	621 (1.9)	4614 (4.7)	822 (1.4)
Urologic	6,292 (1.4)	290 (5.1)	57 (1.2)	643 (1.7)	125 (0.4)	1750 (1.8)	320 (0.5)
Other	45,062 (10.3)	745 (13.1)	502 (10.3)	3715 (10.0)	2256 (6.8)	11170 (11.5)	3873 (6.7)
Unknown	5,983 (1.4)	123 (2.2)	72 (1.5)	479 (1.3)	426 (1.3)	1205 (1.2)	779 (1.3)
Comorbidity							
Hypertension	37,7857 (87.7)	4000 (70.3)	4082 (84	30266 (81.4)	29761 (90.1)	81034 (83.4)	52572 (90.3)
Diabetes	253,851 (58.9)	998 (17.5)	1394 (28.7	17971 (48.4)	16056 (48.6)	58786 (60.5)	35540 (61.1)
Cardiac failure	122,460 (28.4)	305 (5.4)	486 (10.0)	5182 (13.9)	6751 (20.4)	24613 (25.3)	16563 (28.5)
Tobacco use	35,245 (8.2)	428 (7.5)	327 (6.7	4368 (11.8)	2982 (9.0)	10262 (10.6)	5627 (9.7)
Drug abuse	7,241 (1.7)	246 (4.3)	133 (2.7	1406 (3.8)	1094 (3.3)	1572 (1.6)	1916 (3.3)

Atherosclerotic heart disease	54,455 (12.6)	40 (0.7)	50 (1.0)	1840 (5.0)	1281 (3.9)	11698 (12.0)	4850 (8.3)
Peripheral vascular disease	40,699 (9.4)	70 (1.2)	77 (1.6)	1972 (5.3)	1371 (4.2)	10003 (10.3)	4243 (7.3)
Cerebrovascular disease	38,589 (9.0)	66 (1.2)	85 (1.7)	1695 (4.6)	1777 (5.4)	7944 (8.2)	6106 (10.5)
Other cardiac disease							
Cancer	31,191 (7.2)	59 (1.0)	30 (0.6)	885 (2.4)	479 (1.5)	6025 (6.2)	2505 (4.3)
Alcohol abuse	80,56 (1.9)	73 (1.3)	32 (0.7)	1065 (2.9)	549 (1.7)	2455 (2.5)	1557 (2.7)
Chronic obstructive pulmonary disease	44,635 (10.4)	31 (0.5)	31 (0.6)	1349 (3.6)	821 (2.5)	10486 (10.8)	4352 (7.5)
Pre-kidney failure nephrology care[e]							
No	80,793 (18.4)	1339 (23.5)	1468 (30.2)	7504 (20.2)	8782 (26.6)	17378 (17.9)	12643 (21.7)
Yes	288,600 (65.7)	3482 (61.2)	2617 (53.9)	24234 (65.2)	18622 (56.4)	65195 (67.1)	35045 (60.2)
Neighborhood poverty (% of ZIP code residents below poverty)[f,g]							
0%-19.9% below poverty	366,389 (83.4)	5119 (90.0)	3352 (69.0)	33044 (88.9)	22818 (69.1)	87109 (89.6)	39509 (67.9)
≥20% below poverty	68,232 (15.5)	517 (9.1)	1467 (30.2)	3737 (10.1)	9833 (29.8)	8982 (9.2)	17890 (30.7)
**Dialysis Facility-Level Characteristics**[h]							
Profit Status[i]							
For-profit	5,124 (1.2)	206 (3.6)	103 (2.1)	855 (2.3)	642 (1.9)	1502 (1.5)	764 (1.3)
Non-profit	429,256 (97.8)	5293 (93)	4660 (95.9)	35539 (95.6)	31811 (96.3)	94264 (97)	56715 (97.5)
Patient-to-Staff Ratio, mean (SD)[j]	46.9 (7.5)	46.3 (15.9)	46.5 (12.0)	46.9 (10.2)	47.1 (12.0)	47.0 (8.1)	46.9 (9.0)

[a] Data shown as the total number of patients (No.) and the percentage (%), unless indicated otherwise.
[b] Insurance information missing for 16630 patients (3.78%)
[c] Body-mass index calculated as weight in kilograms divided by height in meters squared, missing for 10301 patients (2.34%)
[d] Patient attributable cause missing for 8069 patients (1.84%)
[e] Nephrology care information missing for 70062 (15.94%)
[f] Obtained from American Community Survey Data, 2015-2019.
[g] Information on neighborhood poverty missing for 4834 (1.10%)
[h] Obtained from data from the CMS ESRD Annual Facility Survey and the CDC Dialysis Surveillance Survey within the USRDS facility dataset
[i] Information on dialysis facility profit status missing for 495 patients (0.1%) and unknown for 4580 patients (1.0%)

[j] Number of patients for every 1 social worker. Information on dialysis facility patient-to-social worker ratio was missing for 69236 patients (15.75%). Calculated only among those facilities that have social workers and whose information on number of patients and social workers was not missing

Table S2. Crude Incidence of Waitlisting and Death among kidney failure patients by race and age, 2015-2019, followed through 2020

	**All Patients**			**Non-Hispanic Black**			**Non-Hispanic White**		
	Waitlisting N (%)	Death N (%)	Total No.	Waitlisting N (%)	Death N (%)	Total No.	Waitlisting N (%)	Death N (%)	Total No.
All Ages	82180 (18.7)	164883 (37.5)	439455	26964 (18.0)	45600 (30.4)	150238	55216 (19.1)	119283 (41.2)	289217
Age									
18-29	5264 (49.9)	1272 (12.1)	10548	1878 (38.7)	694 (14.3)	4859	3386 (59.5)	578 (10.2)	5689
30-49	24433 (34.8)	13198 (18.8)	70181	9965 (30.2)	5744 (17.4)	33017	14468 (38.9)	7454 (20.1)	37164
50-64	34628 (22.3)	49281 (31.7)	155385	11222 (19.3)	16231 (27.9)	58195	23406 (24.1)	33050 (34.0)	97190
65-80	17855 (8.8)	101132 (49.7)	203341	3899(7.2)	22931 (42.3)	54167	13956 (9.4)	78201 (52.4)	149174

Abbreviations: N (%), total number of patients (N) and the percentage (%).
Non-Hispanic, White patients are reference group.

Table S3. Incidence of Kidney Failure Patients Placement on the Waitlist During Follow-up by Race and Age, Censoring for Death, 2015-2019, followed through 2020[a]

	Patient Race		
	**All Patients**	**Non-Hispanic Black**	**Non-Hispanic White**
	CIF (95% CI)	CIF (95% CI)	CIF (95% CI)
All Ages	20.6% (20.4, 20.7)	20.7% (20.5, 20.9)	20.5% (20.3, 20.6)
Age			
18-29	55.0% (53.9, 56.1)	44.7% (43.0, 46.4)	63.8% (62.3, 65.2)
30-49	38.7% (38.3, 39.1)	35.0% (34.4, 35.7)	41.9% (41.3, 42.5)
50-64	24.5% (24.3, 24.8)	22.2% (21.8, 22.6)	25.9% (25.6, 26.2)
65-80	9.3% (9.2, 9.5)	8.0% (7.7, 8.2)	9.8% (9.7, 10.0)

Non-Hispanic, White patients are reference group. CI, confidence interval; CIF, cumulative incidence function
[a]Calculated using cumulative incidence function and adjusted for competing risk of death

Table S4. Relative Adjusted Hazard of Waitlisting (non-Hispanic Black vs non-Hispanic White) Among Patients With Kidney Failure 18-29, 2015-2019, followed through 2020[a]

	Patient Race			
	Non-Hispanic Black Patients	Non-Hispanic White Patients		
	Waitlisting (%)	Waitlisting (%)	Unadjusted HR (95% CI)	aHR (95% CI)[a]
Sex				
Male	946 (40.0)	1940 (60.5)	0.50 (0.42, 0.59)	0.68 (0.60, 0.77)
Female	932 (37.3)	1446 (58.3)	0.51 (0.43, 0.61)	0.65 (0.53, 0.74)
Insurance type				
Medicaid	789 (33.0)	861 (43.4)	0.68 (0.59, 0.78)	0.76 (0.67, 0.87)
Medicare	69 (35.0)	107 (54.3)	0.46 (0.35, 0.61)	0.60 (0.45, 0.80)
Private	589 (59.7)	1601 (80.5)	0.50 (0.41, 0.60)	0.59 (0.49, 0.70)
Other	208 (48.3)	506 (67.5)	0.55 (0.44, 0.68)	0.62 (0.49, 0.78)
No coverage	208 (26.9)	192 (36.6)	0.64 (0.51, 0.79)	0.64 (0.50, 0.84)
Body-mass index				
<18	99 (39.6)	223 (58.2)	0.58 (0.46, 0.73)	0.65 (0.50, 0.83)
18-24.9	677 (41.6)	1450 (62.3)	0.54 (0.48, 0.61)	0.66 (0.60, 0.72)
25-29.9	432 (40.5)	755 (61.3)	0.50 (0.42, 0.60)	0.63 (0.51, 0.77)
≥30	648 (35.5)	832 (55.9)	0.48 (0.38, 0.61)	0.63 (0.51, 0.77)
Attributed cause of kidney failure				
Diabetes	335 (28.7)	300 (33.9)	0.83 (0.67, 1.03)	1.01 (0.90, 1.1)
Hypertension	482 (35.3)	441 (51.5)	0.55 (0.45, 0.68)	0.62 (0.50, 0.76)
Glomerulonephritis	782 (51.1)	1408 (69.4)	0.56 (0.45, 0.70)	0.63 (0.52, 0.76)
Polycystic kidney	65 (63.1)	432 (79.4)	0.62 (0.49, 0.79)	0.67 (0.56, 0.80)
Urologic	35 (61.4)	218 (75.2)	0.77 (0.64, 0.93)	0.91 (0.74, 1.13)
Other	138 (27.5)	408 (54.8)	0.38 (0.27, 0.53)	0.43 (0.32, 0.58)
Unknown	28 (38.9)	80(65.0)	0.44 (0.26, 0.75)	0.50 (0.25, 1.0)
Pre-kidney failure nephrology care				
No	468 (31.9)	664 (49.6)	0.53 (0.46, 0.62)	0.64 (0.56, 0.74)
Yes	1177 (45.0)	2315 (66.5)	0.51 (0.43, 0.61)	0.67 (0.58, 0.77)

Neighborhood poverty (% of ZIP code residents below poverty)[e]				
0%-19.9% below poverty	1396 (41.6)	3125 (61.0)	0.52 (0.45, 0.61)	0.63 (0.55, 0.73)
≥20% below poverty	468 (31.9)	228 (44.1)	0.65 (0.54, 0.78)	0.81 (0.68, 0.96)

Abbreviations: CI, confidence interval; HR, hazard ratio.

[a] Cox models were performed to obtain hazard ratios adjusting for age, sex, insurance type at kidney failure onset, body-mass index, attributed cause of kidney failure (diabetes, hypertension, glomerulonephritis, polycystic kidney, urologic, unknown), atherosclerotic heart disease, cardiac failure, peripheral vascular disease, cerebrovascular disease, hypertension, pre- kidney failure nephrology care, diabetes, tobacco use, cancer, chronic obstructive pulmonary disease, drug abuse, alcohol abuse, neighborhood poverty.

Table S5. Association between Race and Waitlisting Among Incident Dialysis Patients (2015-2019) Accounting for Death as a Competing Risk, by Age, followed through 2020

	Unadjusted SHR (95% CI)	Adjusted SHR (95% CI)[a,b]
All Ages	0.92 (0.82, 1.03)	0.88 (0.79, 0.99)
Age		
18-29	0.54 (0.43, 0.67)	0.74 (0.62, 0.88)
30-49	0.71 (0.61, 0.83)	0.90 (0.78, 1.04)
50-64	0.77 (0.68, 0.87)	0.91 (0.83, 1.01)
65-80	0.76 (0.66, 0.87)	0.85 (0.75, 0.96)

Non-Hispanic, White patients are reference group. CI, confidence interval; SHR, subdistribution hazard ratios

[a]Cox models were performed to obtain hazard ratios adjusting for age, sex, insurance type at kidney failure onset, body-mass index, attributed cause of kidney failure (diabetes, hypertension, glomerulonephritis, polycystic kidney, urologic, unknown), atherosclerotic heart disease, cardiac failure, peripheral vascular disease, cerebrovascular disease, hypertension, diabetes, tobacco use, cancer, chronic obstructive pulmonary disease, drug abuse, alcohol abuse, neighborhood poverty.

[b]There were missing data for 82,239 patients (18.7%)

Table S6. Baseline Characteristics of Incident U.S. Adult Patients With Kidney Failure (2015-2019) by Preemptively Waitlisted Status[a] (N=439,455)

	Preemptively Waitlisted (n=32,816)		Non-Preemptively Waitlisted (n=406,639)	
	Non-Hispanic White (n=25,543)	Non-Hispanic Black (n=7,273)	Non-Hispanic White (n=263,674)	Non-Hispanic Black (n=142,965)
**Patient-Level Characteristics**				
Sex				
Male	15,448 (60.5)	3,915 (53.8)	157,406 (59.7)	77,422 (54.2)
Female	10,095 (39.5)	3,358 (46.2)	106,268 (40.3)	65,543 (45.8)
Age				
18-29	1,271 (5.0)	332 (4.6)	4418 (1.7)	4,527 (3.2)
30-49	5,888 (23.1)	2,193 (30.2)	31,276 (11.9)	30,824 (21.6)
50-64	10,602 (41.5)	3,187 (43.8)	86,588 (32.8)	55,008 (38.5)
65-80	7,782 (30.5)	1,561 (21.5)	141,392 (53.6)	52,606 (36.8)
Insurance type[b]				
Medicaid	2,256 (8.8)	1,400 (19.2)	54,131 (20.5)	54,131 (37.9)
Medicare	6,882 (26.9)	1,504 (20.7)	123,212 (46.7)	44,769 (31.3)
Private	12,133 (47.5)	3,257 (44.8)	46,187 (17.5)	24,764 (17.3)
Other	2,324 (9.1)	700 (9.6)	21,103 (8.0)	11,548 (8.1)
No coverage	168 (0.7)	85 (1.2)	8,811 (3.3)	9,229 (6.5)
Body-mass index[c]				
<18	463 (1.8)	104 (1.4)	6,895 (2.6)	4,480 (3.1)
18-24.9	6,171 (24.2)	1,504 (20.7)	61,335 (23.3)	35,699 (25)
25-29.9	7,834 (30.7)	2,261 (31.1)	68,771 (26.1)	36,854 (25.8)
≥30	9,441 (37)	3,116 (42.8)	120,689 (45.8)	63,537 (44.4)
Attributed cause of kidney failure [d]				
Diabetes	6,111 (23.9)	6,111 (84.0)	128,801 (48.8)	63,686 (44.5)
Hypertension	4,615 (18.1)	2,524 (34.7)	65,064 (24.7)	55,533 (38.8)
Glomerulonephritis	4,610 (18)	1,199 (16.5)	18,504 (7)	8,142 (5.7)
Polycystic kidney	5,166 (20.2)	545 (7.5)	5,894 (2.2)	1,426 (1)
Urologic	587 (2.3)	51 (0.7)	4,853 (1.8)	801 (0.6)
Other	2,592 (10.1)	380 (5.2)	32,260 (12.2)	9,830 (6.9)
Unknown	462 (1.8)	115 (1.6)	3,491 (1.3)	1,915 (1.3)
Comorbidity				
Hypertension	20,524 (80.4)	6,421 (88.3)	221,973 (84.2)	128,939 (90.2)
Diabetes	7,650 (29.9)	2,930 (40.3)	15,8713 (60.2)	84,558 (59.1)
Cardiac failure	1,443 (5.6)	683 (9.4)	79,482 (30.1)	40,852 (28.6)
Tobacco use	543 (2.1)	178 (2.4)	22,758 (8.6)	11,766 (8.2)
Drug abuse	44 (0.2)	23 (0.3)	3,537 (1.3)	3,637 (2.5)
Atherosclerotic heart disease	1,581 (6.2)	328 (4.5)	40,027 (15.2)	12,519 (8.8)
Peripheral vascular disease	781 (3.1)	223 (3.1)	29,258 (11.1)	10,437 (7.3)
Cerebrovascular disease	726 (2.8)	288 (4.0)	23,145 (8.8)	14,430 (10.1)
Other cardiac disease	2,437 (9.5)	609 (8.4)	61,232 (23.2)	23,002 (16.1)
Cancer	1,133 (4.4)	247 (3.4)	22,403 (8.5)	7,408 (5.2)
Alcohol abuse	176 (0.7)	26 (0.4)	5,028 (1.9)	2,826 (2.0)
Chronic obstructive pulmonary disease	464 (1.8)	122 (1.7)	33,375 (12.7)	10,674 (7.5)
Pre- kidney failure nephrology care[e]				
No	611 (2.4)	286 (3.9)	47,971 (18.2)	31,925 (22.3)
Yes	21,845 (85.5)	6,134 (84.3)	175,995 (66.7)	84,626 (59.2)
Neighborhood poverty (% of ZIP code residents below poverty)[f,g]				

0%-19.9% below poverty	24,245 (94.9)	5548 (76.3)	23,8347 (90.4)	98,249 (68.7)
≥20% below poverty	1,028 (4.0)	1,628 (22.4)	22,573 (8.6)	43,003 (30.1)
**Dialysis Facility-Level Characteristics**[h]				
Profit Status[i]				
For-profit	22,649 (88.7)	6,448 (88.7)	25,9771 (98.5)	140,388 (98.2)
Non-profit	1,384 (5.4)	438 (6.0)	1,997 (0.8)	1,305 (0.9)
Patient-to-Staff Ratio, mean (SD)[j]	42.5 (39.9)	3915 (53.8)	47.0 (5.2)	47.0 (7.2)

[a] Data shown as the total number of patients (No.) and the percentage (%), unless indicated otherwise.
[b] Insurance information missing for 16630 patients (3.78%)
[c] Body-mass index calculated as weight in kilograms divided by height in meters squared, missing for 10301 patients (2.34%)
[d] Patient attributable cause missing for 8069 patients (1.84%)
[e] Nephrology care information missing for 70062 (15.94%)
[f] Obtained from American Community Survey Data, 2015-2019.
[g] Information on neighborhood poverty missing for 4834 (1.10%)
[h] Obtained from data from the CMS ESRD Annual Facility Survey and the CDC Dialysis Surveillance Survey within the USRDS facility dataset
[i] Information on dialysis facility profit status missing for 495 patients (0.1%) and unknown for 4580 patients (1.0%)
[j] Number of patients for every 1 social worker. Information on dialysis facility patient-to-social worker ratio was missing for 69236 patients (15.75%). Calculated only among those facilities that have social workers and whose information on number of patients and social workers was not missing

Table S7. Incidence of Kidney Failure Patients Placement on the Waitlist During Follow-up by Race and Age, Excluding Preemptively Waitlisted Patients and Treating Death As a Competing Risk, 2015-2019, followed through 2020[a]

	Patient Race		
	**All Patients**	**Non-Hispanic Black**	**Non-Hispanic White**
	CIF (95% CI)	CIF (95% CI)	CIF (95% CI)
All Ages	14.1% (14.0, 14.3)	16.7% (16.4, 16.9)	12.8% (12.6, 12.9)
Age			
18-29	47.0% (45.7, 48.2)	40.7% (38.9, 42.5)	53.4% (51.6, 55.1)
30-49	30.7% (30.3, 31.1)	30.4% (29.8, 31.1)	31.0% (30.4, 31.6)
50-64	17.2% (17.0, 17.4)	17.7% (17.3, 18.1)	16.9% (16.6, 17.1)
65-80	5.0% (4.8, 5.1)	5.2% (5.0, 5.5)	4.8% (4.7, 5.0)

Non-Hispanic, White patients are reference group. CI, confidence interval.
[a]Calculated using cumulative incidence function and adjusted for competing risk of death
